# Insulinoma in pediatric tuberous sclerosis complex: a case report

**DOI:** 10.3389/fped.2023.1216201

**Published:** 2023-07-19

**Authors:** Katia Librandi, Serena Grimaldi, Silvia Catalano, Francesco Moro, Stefano Gabriele Vallero, Marco Spada, Francesco Porta

**Affiliations:** ^1^ Postgraduate School of Pediatrics, University of Turin, Turin, Italy; ^2^ Department of Nuclear Medicine, University of Turin, Turin, Italy; ^3^ General Surgery 2U, University of Turin, Turin, Italy; ^4^ Department of Pediatrics, University of Turin, Turin, Italy

**Keywords:** tuberous sclerosis complex (TSC), insulinoma, TSC, oncology, PNET

## Abstract

**Background:**

Tuberous sclerosis complex (TSC) is a rare multisystemic disorder. This genetically determined disease is characterized by highly variable clinical expression, including epilepsy as a common feature. Seizures can also occur as a manifestation of symptomatic hypoglycemia. The latter could be caused by an insulinoma, whose association to TSC has already been debated. In TSC-associated tumors, dysregulation of the mTOR pathway is believed to be present, leading to significant impacts on cellular metabolism, growht, and proliferation. To date, the association between TSC and insulinoma has been reported in 11 adults. Here, we present the first case of a pediatric patient with TSC diagnosed with an insulinoma and review the existing literature on this topic.

**Case presentation:**

A 11-year-old female with TSC presented with seizures unresponsive to standard therapy. Further investigation revealed that these seizures were caused by hypoglycemia. Subsequent evaluation led to the diagnosis of a pancreatic insulinoma, which was surgically removed. Following the procedure, the patient was free from seizures.

**Conclusions:**

In individuals with TSC, the recurrence of epileptiform episodes throughout their lifetime, especially if previously well controlled with antiepileptic therapy, should raise suspicion for hypoglycemic events. These events may potentially be associated with the presence of an insulinoma. Further research and increased awareness are necessary to gain a better understanding of the association between TSC and insulinomas, and to guide clinical management strategies.

## Background

Tuberous sclerosis complex (TSC) is an autosomal dominant genetic disorder due to mutations in the TSC1 or TSC2 genes, with an incidence of 1 in 5,000–10,000 live births. Clinically, TSC has been known since the mid nineteenth century as a multisystem disease. The main features of TSC include epilepsy, intellectual disability, and adenoma sebaceum (known as Vogt’s triad). After the first depiction of the typical TSC skin lesions by Rayer in 1835, association with multi-localized tubers was successively described by von Recklinghausen (1862) and Bourneville (1880), and TSC was classified as a phacomatosis by van der Hoeve in 1933 (1). TSC1 and TSC2 genes encode hamartin and tuberin, respectively. These proteins function as a complex regulating the activity of the mechanistic target of rapamycin (mTOR) kinase and exerting effects on cell proliferation and autophagy. In TSC, abnormalities in the hamartin-tuberin complex lead to the development of various benign and malignant tumors, including angiofibromas, retinal hamartomas, subependymal nodules (SENs), cardiac rhabdomyomas, lymphangioleiomyomatosis, angiomyolipomas, and subependymal giant cell astrocytoma (2). Pancreatic neuroendocrine tumors (pNETs) have been anecdotally reported in 11 adult patients with TSC. In this report, we present the first TSC-related insulinoma in pediatrics and we review the literature on this topic.

## Case presentation

**Figure 1:**
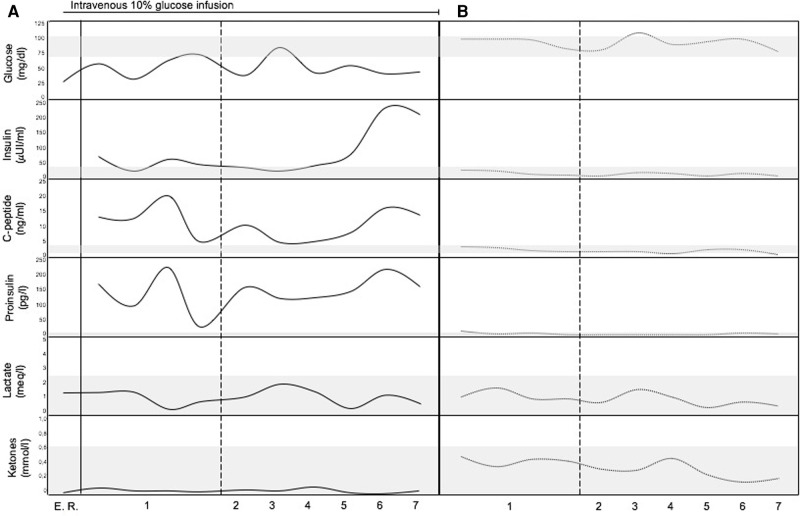
Metabolic profile of the patient. (**A**) Before surgical treatment (continuous lines). (**B**) After surgical treatment (dotted lines). The gray bands represent normal range of the analytes (vertical axis). Horizontal axis represents time (numbers refers to the days before and after surgical removal of the insulinoma). E. R., Emergency Room.

An 11-year-old female patient with TSC presented a medical history of levetiracetam-responsive epilepsy since the age of 1 year, associated with multiple cortical tubers, cardiac rhabdomyomas, hepatic and renal angiomyolipomas, and iris hamartomas. Genetic analysis identified a heterozygous, *de novo* mutation (c. 1444-1G > A) in the TSC2 gene, which has been previously described (3). During a routine morning-fasting blood sampling for biochemical monitoring, the patient experienced an acute episode characterized by stereotyped and jerky movements of the upper limbs, followed by drowsiness. The patient was admitted to the Emergency Room. The external body temperature was 36.3°C, and the general condition of the patient was good. No signs of cutaneous or mucosal bleeding were observed. Slurred speech and fine tremors of the upper extremities were reported, with no other neurological signs or symtpoms present. Cardiopulmonary and abdominal examinations yielded normal findings. Blood testing revealed isolated hypoketotic hypoglycemia, with a glucose level of 26 mg/dl (1.4 mmol/L), below the normal range of 70–100 mg/dl (3.9–5.6 mmol/L). The ketone level was 0.06 mg/dl (0.01 mmol/L), within the normal range of 0–0.34 mg/dl (0–0.6 mmol/L). Immediate administration of a 3 ml/kg bolus of intravenous 10% glucose solution over a 10-minute period resulted in a rapid restoration of normal consciousness. This was followed by a continuous infusion of the same glucose solution at a rate of 20 ml/h. Electroencephalography (EEG) results were normal. A comprehensive medical history revealed a pattern of frequent post-prandial confusion, inappropriate verbal responses, dysarthria, and drowsiness that had been ongoing for approximately two years. A 72-hour fasting test was initiated. Intermittent blood glucose monitoring during the fasting test revealed alternating episodes of hypoglycemia, accompanied by distinct metabolic features consistent with hyperinsulinism (Figure 1A). Insulin and C-peptide levels were measured—simultaneously with glicemia—on five occasions, including two measurements during fasting and three after meals. The fasting test had to be discontinued due to significant symptomatology.

**Figure 2:**
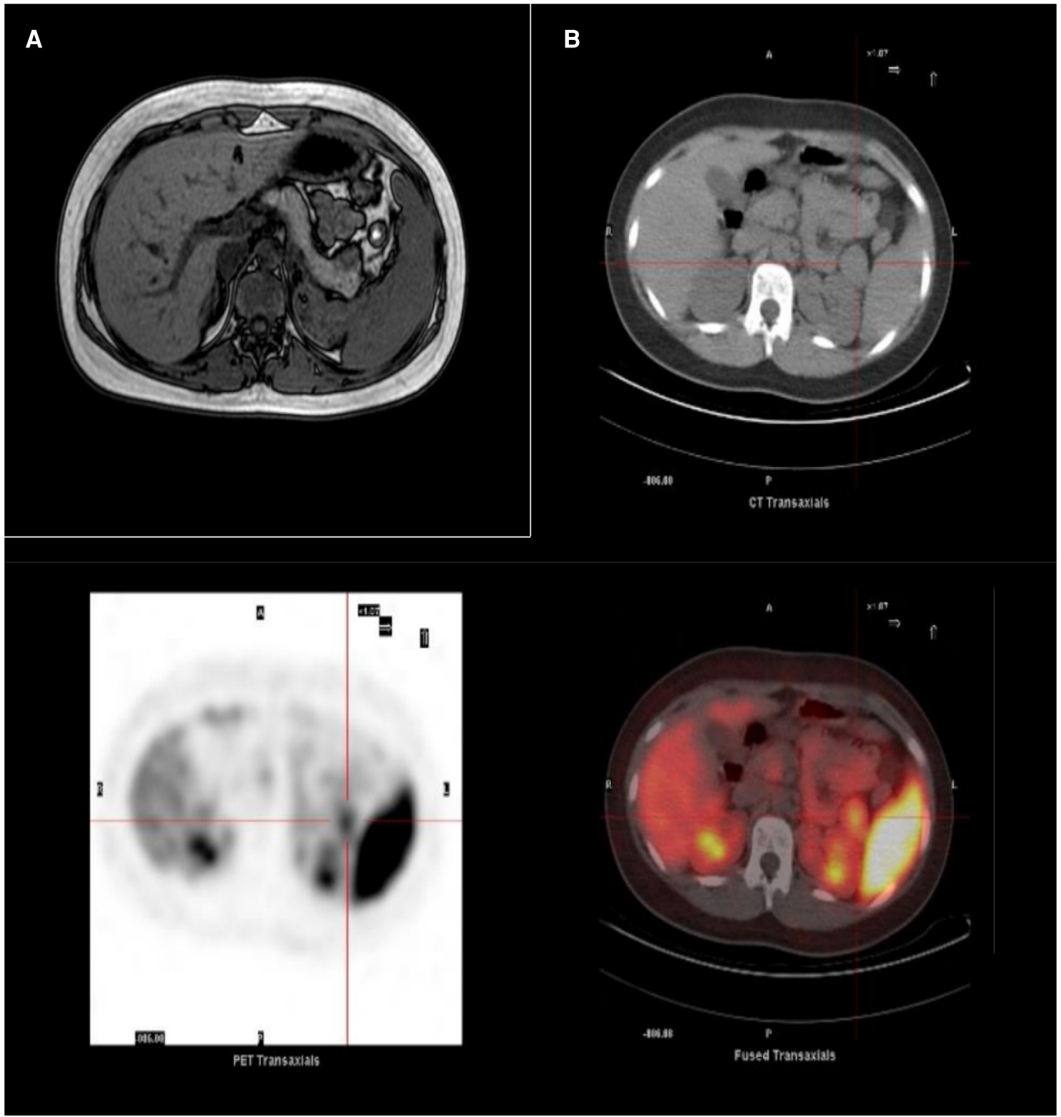
Imaging of insulinoma. (**A**) MRI showing a lesion in the pancreatic tail. (**B**) ^68^Ga-DOTATOC PET/CT scan showing selective radiotracer uptake by the lesion consistent with insulinoma.

An abdominal multiphase computed tomography (CT) scan revealed an arterial phase enhancing lesion (maximum diameter 29 mm) at the pancreas tail, which was further confirmed by magnetic resonance imaging (MRI). ^68^Ga-DOTATOC positron emission tomography/computed tomography (PET/CT) imaging demonstrated selective radiotracer uptake by the lesion, consistent with insulinoma (Figures 2A,B). Laparoscopic distal pancreatectomy allowed the excision of the insulinoma and the full correction of clinical and metabolic picture (Figure 1B). Pathological examination revealed an organoid growth of a well-differentiated tumor, with no signs of necrosis or vascular invasion. Neither perineural infiltration nor infiltration of the peripancreatic adipous tissue was observed. The mitotic count was 6/10 per High Power Field. Immunohistochemical staining showed positive results for cromogranine A and insulin. The proliferation index, evaluated with anti-Ki67 coloration, was 10%. The diagnosis was a pNET G2 with positivity for insuline, classified as pT2 according to the UICC VIII ed. 2017 staging system. After surgery the patient had no recurrence of seizures at 18 months of follow up.

## Discussion

In TSC, epilepsy is a commonly observed feature, while insulinoma is a rare occurence. Epilepsy serves as a key diagnostic indicator of Vogt’s triad, whereas the association between TSC and insulinoma has been documented in only 11 adult patients (Table 1). Insulinoma presents clinically with the Whipple’s triad, characterized by hypoglycemia, neuroglycopenic symptoms (including sympathoadrenal symptoms, confusion, unusual behavior, visual changes, cephalgia, and seizures), and symptom resolution after restoration of normoglycemia.

**Table 1:** Review of the case reports of patients with tuberous sclerosis complex and insulinoma.

Reference	Gender	Age at diagnosis (years)	Localization of insulinoma	Maximum diameter of insulinoma (mm)	Clinical findings leading to diagnosis
Gutman and Leffkowitz (4)	Female	24	Pancreas (body)	30	Seizures beginning with agitation and groaning, evolving in clonic movements of the extremities and utmost rigidity. Abnormalities of sleep-wake cycle. Slurred speech. Excessive sweetening of tea beverages
Davidson (5)	Female	21	Pancreas (unspecified)	Not available	Seizures, outburst of aggressive behaviour, confusion with psychomotor agitation and myoclonic twitching
Davoren and Epstein (6)	Male	23	Pancreas (head)	30	Seizures. Sleepiness after exertion. Increased appetite for sweet foods
Kim et al. (7)	Male	28	Pancreas (tail)	20	Behavioral changes characterized by episodes of agitation and, other times, lethargy
Simon et al. (8)	Female	19	Pancreas (tail)	18	Behavioral changes characterized by episodes of agitation and aggressiveness. Hypoglycemic coma
Boubaddi et al. (9)	Female	18	Pancreas (unspecified)	Not available	Unspecified “symptomatic hypoglycemia”
Eledrisi et al. (10)	Male	43	Bulk in the abdomen	210	Mental confusion, slurred speech and abnormal behavior. Episodes of sweating and dizziness
Le Berre et al. (11)	Male	41	Pancreas (tail)	30	Seizures with loss of consciousness
Kang et al. (12)	Male	23	Pancreas (tail)	35 (tumor 1) 7 (tumor 2)	Altered behavior and increasing seizure frequency
Al Qahtani et al. (13)	Male	47	Pancreas (head)	20	Incidental finding during routine follow-up
Piskinpasa et al. (14)	Male	32	Pancreas (tail)	12	Palpitations, diaphoresis, confusion
Present case	Female	11	Pancreas (tail)	29	Seizures beginning with agitation, mental confusion, slurred speech, evolving in clonic movements of the extremities. Dizziness and sleepiness. Great hunger.

The table reports the 11 cases of insulinoma in patients with tuberous sclerosis complex. The twelfth case is that of our study. In the first column the authors and references of case reports are reported. The second column reports the gender of the patients, the third column the age, the fourth the precise localization of the insulinoma, the fifth column the maximum diameter of the lesion. The sixth column describes the symptomatology leading to the diagnosis of insulinoma.

The pathophysiology of neuroendocrin tumors NETs in TSC patients involves a dysregulation of cellular signaling pathways and loss of function in the TSC1 and TSC2 genes, leading to hyperactivation of the mTOR pathway. This disruption of cellular processes, including cell growth and metabolism, contributes to the development of insulinomas (15). Notably, the occurrence of insulinomas has been particularly associated with TSC2 (tuberin) inhibition (12).

One notable distinction between TSC-associated insulinomas and sporadic cases is that the diagnosis of TSC-associated insulinomas occurs at a younger age and with larger tumor sizes (16). However, the clinical presentation of both categories is largely similar. The clinical features of insulinoma in TSC can differ in adult and pediatric population. In adult cases, seizures are often preceeded by behavioural changes (agitation, groaning, outburst of aggressiveness). In our pediatric patient, agitation was observed prior to the onset of the seizure, although no aggressiveness was noted. Slurred speech, mental confusion, dizziness, and sleepiness are commonly reported in adult cases and were also present in our pediatric case. In children, difficulty expressing their needs (e.g., increased appetite, especially for sweet foods) could contribute to a delay in diagnosis. In our case report the suspicion of a hypoglycemic state arose from the presence of typical neuroglycopenic and autonomic symptoms. The absence of acidemia, low free fatty acids, and hypoketonemia, coupled with high levels of insulin and C-peptide during hypoglycemia, confirmed the diagnosis of hyperinsulinism. Imaging studies revealed the lesion in the pancreas, which is the most common site of development. We took advantage of the characteristic overexpression of somatostatin receptors (SSTRs), predominantly subtype 2 (SSTR2), in most neuroendocrine tumors (NETs) using a radiolabeled somatostatin analogue (^68^Ga-DOTATOC) suitable for PET imaging. Through this approach, we successfully diagnosed the first reported case of insulinoma in a pediatric patient with TSC. The median age at diagnosis of insulinoma in TSC is 27.5 years, and all reported cases have originated in the pancreas. In six out of twelve cases (including the newly reported patient), the pNET was found in the pancreatic tail. The maximum reported diameter of insulinomas was 21 cm. Clinical manifestations of insulinoma in adult TSC patients, such as seizures and behavioral changes, were also observed in our patient. Recurrent symptoms of sleepiness, slurred speech, and confusion, as experienced by our patient, were frequently reported as well.

## Conclusion

Based on our case report and thorough literary review, we propose that the recurrence of epileptiform episodes throughout the lifetime of TSC patients, especially when previously well controlled by antiepileptic therapy, should raise suspicion of hypoglycemic events potentially associated with insulinomas. Hypoketotic hypoglycemia serves as a metabolic hallmark of hyperinsulinism. Although rare in TSC, insulinoma represents a treatable cause of hypoglycemia. Therefore, we strongly recommend that physicians consider insulinoma as a possible etiology of seizures in patients affected by TSC. To facilitate early detection, we suggest including a 72-hours fasting test in the follow-up protocol for all TSC patients, especially those presenting with recurrent seizures that are no longer controlled by a long-standing antiepileptic therapy, and in the absence of changes in EEG tracing or brain imaging explaining new epileptic onsets.

## Data Availability

The raw data supporting the conclusions of this article will be made available by the authors, without undue reservation.
